# An Essay on the Remittent and Intermittent Diseases, Including, Generally Marsh Fever and Neuralgia

**Published:** 1830

**Authors:** 


### REVIEWS AND BIBLIOGRAPICAL NOTICES.

---

ART. X.—*An Essay on the remittent and intermittent diseases, including, generically Marsh Fever and Neuralgia. Comprising, under the former, various anomalies, obscurities, and consequences, and, under a new systematic view of the latter, treating of Tic Douloureux, Sciatica, Headach, Ophthalmia, Toothach, Palsy, and many other modes and consequences of this generic disease.* By JOHN MACCULLOCH, M. D., F. R. S., ETC. ETC. Physician in ordinary to his Royal Highness Prince Leopold of Saxe Cobourg.—pp. 474.

Our last number contains an extended analytical review of Dr. M'Cullough's Essay on the production and propagation of Malaria. We propose in the present, to analyze his treatise on the diseases produced, or supposed to be produced, by this occult cause. These, according to our author, are neither few nor feeble, as will appear from the following systematic catalogue ; which, although, placed at the end of his book, we choose to prefix to our review, that the reader may have a full understanding of what Dr. M. proposes to establish.

### TABULAR VIEW.

*Of the Diseases appertaining to Marsh Fever and Neuralgia.*

---

### MARSH FEVER.

CONTINUOUS }
REMITTENT }

Ephemera }
Synocha }
Synochus }

SINGLE OR TERMINATING
CHRONIC OR RELAPSING
ANOMALOUS AND SIMULATING : *vide* Intermittent.

INTERMITTENT
QUOTIDIAN
——— Double
TERTIAN
——— Double
QUARTAN
——— Double
——— Triple
QUINTAN, &c.
SINGLE OR TERMINATING
CHRONIC OR RELAPSING
ANOMALOUS AND SIMULATING. With local symptoms often more conspicuous than febrile ones ; and chiefly so when chronic. Often deceptive in these cases, or mistaken for original or separate diseases.

| Terms from *Authorities.* | PARALYTIC. | |
|---|---|---|
| Carotica } | Apoplexy—primary : repeated | |
| Soporosa } | Lethargy ——— periodical | |
| Comatosa | Coma ——— periodical | |
| | Universal palsy—permanent | |
| Hemiplegica | Hemiplegia | permanent : periodical |
| | Paraplegia | permanent : periodical |
| | Local palsies | permanent : periodical |

SPASMODIC.

| | | |
|---|---|---|
| Epileptica | Epilepsy | irregular : periodical |
| Epileptica | Convulsions | irregular : periodical |
| Cataleptica | Catalepsy : periodical | |
| Hysterica | Hysteria : irregular : periodical | |
| Pertussis | Spasmodic cough : periodical | |
| Asthmatica | Asthma, Dyspnœa : periodical | |
| Stranguriosa | Irritability of bladder and strangury : periodical Palpitation of heart | |

NERVOUS MISCELLANEOUS.

| | | |
|---|---|---|
| Amens. Amentia quartana | Mania | permanent : periodical |
| | Fatuity | permanent : periodical |
| Hypochondriaca | Hypochondriasis, permanent : periodical | |
| | Vertigo, periodical : transitory | |
| Cephalalgica | Headachs, irregular : periodical | |
| | Dyspepsia, irregular : periodical | |
| | Deafness, periodical | |
| | Amaurosis, periodical : durable | |
| | Nervous disorders : undefinable | |
| | Chlorosis | |

Morbus Pannonicus Debility
Atrophy

---

Emetica ............... Vomiting, periodical, chronic : acute, irregular
Diarrhœa : periodical : permanent
Menorrhagia, Amenorrhea, Dysmenorrhea
Diabetes

INFLAMMATORY.

Pleuritica ............ Pleurisy, permanent : periodical
............ Hepatitis
............ Rheumatism

Arthritica ............................ general, local, acute, chronic, of joints } permanent : periodical of intercostal muscles : false plu'sy

Nephralgica ......... Nephralgia
Catarrh : permanent acute : periodical, chronic

Ischiatica ............ Sciatica, periodical : permanent. Neuralgia
Phthisis
Hectic, false

Urticata .............. unknown to me, and not intelligible, or doubtful.

DYSENTERY,

Acute
Chronic.

CHOLERA,

Indian Cholera ; if different,
Common Cholera.

### SUPPLEMENT.

### *ENDEMICS SAID TO BE PRODUCED BY MALARIA.*

(MONFALCON AND OTHERS.)

Scurvy
Pellagra
Bronchocele
Ulcers
Rickets
Cretinage
Hernia
Varix
Elephantiasis
Asthma
Angina
Œdema of the lungs
Visceral obstructions : and dropsy, as consequences
Scrofula
Phthisis

### NEURALGIA.

*Intermittent febrile Symptoms marked, obscure, or undiscoverable, or possibly not existing.*

PAINFUL.

OF ASSIGNABLE NERVES. Pain acute : periodical or irregular.

Spinal Marrow. As yet little known or observed.
Nerves of face. Tic douloureux, in any part of the head as well as of the face.
Optic nerve.
—— of teeth. Toothach without caries.
* Sciatic nerve. Sciatica.
Anterior crural, in various parts, with various Neuralgiæ.
Spermatic nerve.
Radial. In various parts, with various Neuralgiæ.
—— Of fingers.
—— Of toes.
&c. &c. &c. Blanks to be filled : records not yet found.

OF UNASSIGNABLE NERVES Pain commonly less acute, or dull, or none : periodical or irregular : very acute however in some cases.

* Headach
Common, confined to parts, or general.
Intermitting.
Hemicrania.
Clavus.
Wandering or unfixed toothach , or face-ach.
* Palpitation of heart. No pain.
Palpitation of aorta. No pain.
Palpitation of cœliac artery ?
Stomach pains ?
Colic ——— ?
* Kidney and ureter. Decided Neuralgia : nephralgic pains.
Bladder and neck. Irritability : strangury ; no pain.
Rectum.
Testicle. Acute pain.
Palus.
Cauda equina. Lumbago.
Dr. Alderson ...... Mamma. Acute pain.
Knee. Pain various
Shin bone. Anterior tibial ? considerable pain.

INFLAMMATORY, with neuralgic pains of assignable or unassignable nerves : acute or dull : irregular or periodical,

* LOCAL RHEUMATISM OF LIMBS.

in muscles.
in joints.

RHEUMATISM OF HEAD.

in face. Rheumatic (inflammatory) headach.
in jaw. Rheumatic toothach.

OPHTHALMIA. Fixed or intermitting.

Acute.
—— suppurating.
—— Inflammation of Iris.
Chronic.
Transferable.

FROM INJURY. With inflammation, or not.

from wounds or punctures of nerves : any where.
—— from blood-letting.
—— from amputation.
from blows : any where.
from ulcers : any where.
—— from caries of teeth : carious toothach.
from tumours, aneurisms, &c.
from corns.

NEURALGIC AFFECTIONS OF GLANDS. Irregular, or periodical.

* Diabetes : periodical.
Lacrymation : general : occasional, or periodical.
—————— Of one eye : false fistula. Periodical : irregular.
* Salivation.
* Diarrhœa. Uncertain : periodical :
—————— tertian :
—————— quotidian.
Semi catarrh. Irregular : periodical.

CONSEQUENCES OF NEURALGIA : during, or subsequent to the painful state.

GENERAL,

* Mania. Irregular : periodical.
—————— tertian :
—————— quotidian.
* Fatuity. Permanent : periodical.
—————— tertian.
* Hemiplegia.
* Nervous diseases. Undefinable in variety and number.

PARTIAL. Local paralytic affections.
numbness : any where.
tingling : sensibility : facility of being affected by pressure,
* Amaurosis. Permanent : periodical.
Contraction of Iris. Permanent : periodical.
Opacity of cornea.
Cataract ?
Caries of teeth ?
Gum boils and abscesses. Irregular : periodical.

N. B. As in the first division of this table, it is supposed that there are blanks yet to be filled in the whole. It cannot be given as a perfect enumeration ; being founded solely on the author's own narrow experience.

The affections marked with * have been unavoidably also enumerated under intermittent, since they occur in both ; or else the exact character of the disease is, under the present separation, unassignable.

When a man of acknowledged talents, after extensive research, and deep reflection, arrives at the conclusion, that this appalling *catalogus morborum*, are the offspring of Malaria ; and calls upon the profession to scrutinize his researches, it is undeniable, that they are bound, by considerations of professional duty, to give the subject a full and fair investigation. Dr. M'Cullough regards himself, and with some justice, as having travelled over untrodden ground ; and, therefore, invites his brethren to observe and reason for themselves. To no portion of them is this call more appropriately made, than to the readers of this Journal ; scattered, as they are, over inhabited parts of the vast region, which extends from the Alleghany to the Chippewan mountains, and from the marshy sources of the Mississippi, to the *bayous* and *lagunes* of its delta, at the Gulf of Mexico. The general inclination of this immense plain, is to the south ; its surface is every where covered with a rank vegetation, annually reproduced to undergo a rapid decay ; the heat of its sun in summer, is almost tropical ; its showers are frequent, and most of its waters, both running and stagnant, are widely bordered with alluvion : Therefore, if the earth *can* send forth exhalations unfriendly to the human constitution, we may expect to meet them, in what is denominated the great valley of the Mississippi. And that it does, in reality give out something noxious, seems established by the single fact, that in situations where moisture, intense heat, a fertile soil, and accumulated vegetable remains, are combined, the inhabitants are more unhealthy, than where either of them is absent. Assuming, then, the fact, that the good people of the west and south breathe an atmosphere tainted with an invisible, and intangible gas, we are constrained to urge upon our brethren a diligent study of its origin and effects. Let it not be supposed, however, that we charge upon our portion of the United States, an exclusive generation of Malaria. Other sections are, equally, prolific in that particular; and the remarks just made are as applicable to the banks of the Potomac and Delaware, as to the Ohio and Missouri.

The first chapter of Dr. M's book treats of the ordinary remittent, or marsh fever. All who believe in the existence of Malaria, regard this disease as one of its most obvious, as it constitutes one of its most formidable effects. Our author, therefore, places it at the head of the great class of diseases originating in Malaria.

"It is, says he, the most important link of the philosophical chain, and, further, it is connected with others as their apparent cause: or, that disordered state of the body which commences as remittent, may terminate in almost any other modification of the diseases of Malaria; probably in all."

On the frequency of this fever, in some of its modifications, Dr. M. speaks as follows:

"All therefore which I can here permit myself to say is, that I have attempted to prove that all the fevers of any moment which are not produced by contagion are the effects of Malaria, very often, perhaps very generally, in our own country overlooked; and that while these two leading classes constitute the great mass of fevers throughout the world, those which arise from the other causes here alluded to, are proportionally very small in number, and of very little moment as diseases, from their trifling power on the body."

When remittent fever has been once excited, however, by its only original cause, it may recur even at distant periods from habit, or the action of other causes.

In the following paragraphs, Dr. M. discusses the question of the comparative liability of native and strangers.

"To pass to other matters, it is a question of some importance what are the comparative effects of Malaria in this case, on the natives and on strangers, or visiters; and it is one of some little intricacy, or on which at least there are many contradictions, real or apparent. Such as these are, they will be generally solved by distinguishing between what, for want of better terms, may be called the chronic and the acute action of Malaria.

What the diseases arising from this poison are, and what their effects on the natives and inhabitants of unhealthy districts, have been detailed in the preceding essay. In this place I must add, that, setting aside what may be called the chronic effects, and the disorders exclusive of fever and dysentery chiefly, it is not the fate of the inhabitants to suffer acutely from the latter in every season; while it is plain that if this were the case, extermination must be the speedy consequence. The more usual cause is, that after one, or perhaps more, severe fevers, this disease becomes chronic and intermittent; persisting uniformly in some cases, and, in others, ceasing during certain periods, whether annual or of longer duration, to be again renewed in a similar manner: a few becoming freed from it after a certain number of years, and at a period of life seldom under fifty, while a greater number carry it to that grave, of which the time ranges for them between thirty-five and fifty, if they have escaped as far as the former period. Such is the nature, and quality, or extent, of this acquired habit of resistance by the natives of an unhealthy district; or, in vulgar language, the "seasoning," which is also acquired by emigrants, if in a much inferior proportion: though I have stated a definite and perfect case, from which very many must necessarily vary, but with which they can be compared.

Thus such seasoned persons, or natives, escape many of the acute fevers which seize on emigrants and visiters; but in seasons of peculiar epidemics, they also suffer similarly; their powers of resistance extending only, as it would seem, to a certain point: and hence the mortalities so often recorded, which mark peculiar years, involving all in a common destruction. These considerations, varied according to climates, countries, or circumstances generally, which it would be tedious to detail, will explain the chief facts here concerned: while among minuter particulars which it would be endless to examine, it must be remarked that in certain climates, intermittent, or a chronic state of fever with facility of recurrence being more rare than in others, such a fever, once undergone, may be the pledge of a long continued security."

We have observed, at Cincinnati, that emigrants are not more liable to remittent fever, than the natives or those who had resided in the city for years. Whether the same thing is true of other Western towns we are not able to say. The great epidemic of Louisville, in 1822, affected many of the native citizens, in common with those who had recently arrived. It does not appear, however, that the native and acclimated inhabitants of New Orleans, are as liable as new comers and travellers.

Dr. M. proceeds to inquire into the time, at which the disease appears after exposure to Malaria, and presents us with the following observations:

"If my own frequent observations show that fever may be induced within half an hour after exposure to Malaria, and that a single inspiration, or the space of a very few seconds, is amply sufficient for the purpose, this is also an opinion most decidedly stated by many French and Italian physicians whose experience and acuteness will not be questioned. It is equally the opinion of other observers, not physicians, and therefore without the bias which might be suspected in such cases: of military, and chiefly of naval men, whose observations have been founded on the momentary and transitory effects of a breeze of wind, and especially of a land wind blowing off to sea. In France and in Italy, to confirm this, instances are known and recorded, of labourers dying instantaneously from merely sitting or lying down on the ground, and of others who from looking into a ditch or drain, have been struck dead by that poison which, of course in a minor degree, would have merely produced a fever. Lind, also, whose authority stands high, describes the instant seizure with nausea and delirium, as many others have done; so that respecting this part of the question there needs be no dispute."

These remarks are followed by others relative to the greatest length of time that the poison may lie dormant in the system, or the febrile developements be postponed, after Malaria has impressed its action on the body.

"The more difficult point to determine is, to how long an interval after its application the action of this poison can be delayed: and here, to quote Lind again, this limit is extended by him as far as twelve days. As I have had occasion to say elsewhere, it is not believed by any one of whom I know, that Malaria can, like the matters of contagion, be attached to a substance of any nature, and thus conveyed to excite its diseases; and the observations necessary to determine this interval are not therefore entangled; while it is plain that, to make them truly, the patients must, after a momentary or brief exposure, be completely removed from all the original causes. It may be questioned whether this has often been carefully done or recollected ; while it is certain that from a very frequent or general neglect of the obscurer spots or causes producing Malaria, such persons may often have been unwarily exposed to them, thus easily leading to unfounded opinions on this subject. And as many of the recorded ones have been derived from facts occurring where armies remained on a given spot of ground, though the individual was not a second time exposed to the more obvious cause, it is evident that fallacy is easily introduced into the observation : particularly when what has been formerly said on the propagation, as well as the production of this poison, is considered.

Whatever the truth may be in this case, the present doubts are not expressed without as ample an examination of evidence as it has been in my power to make, and that examination consisting in attempts to ascertain at what most distant period remitting fever has appeared in ships after leaving the shore and thus getting out of the influence of the land winds. At first sight indeed, the opinion in question would appear to be confirmed by many of the cases which I have obtained from the log-books of ships of war, of which I shall however name but one, as it will be sufficient for the purpose. In this instance, a remittent appeared among the crew when on the coast of Africa ; when the vessel put to sea on a cruize, notwithstanding which, however, other men became sick in succession during the space of twenty days, after which no further cases occurred."

Our author has his doubts, whether the cases of remittent and intermittent fever, which are said, by writers, to have occurred several months after exposure, were not owing to a new application of the cause. The subject is evidently one of difficulty ; but we incline to the opinion which he impugns. We have met with cases of vernal intermittent, the disease having been epidemic the preceding autumn, in persons not then affected, as early as the month of March, when the heat, as we presumed, had not yet been sufficient, to develope Malaria ; and we recollect a case of this disease in a lady, who had the fall before travelled through a malarious district and escaped the disease, while several of her fellow travellers suffered under it. Some of them had relapses about the same time with her original attack. The city, at the time, was so free from this form of fever as to favor the belief that these patients were not taken down from any new action of the poison.

Our author proceeds to treat of the symptoms and terminations of remittent fever. We shall pass over the former in silence. On the latter we may remark, substituting our own observations for his, that we have observed four principal modes of termination :

First—In health, by a crisis or a series of evanishing paroxysms, at the end of a variable period.

Secondly—In intermittent fever, especially that modification which is denominated ague.

Thirdly—In a train of symptoms, to which the epithet typhus mitior, is not inapplicable.

Fourth—In a congestion, subacute inflammation, or neuralgia of a joint or some internal organ.

Of the causes which determine these different modes, we are not prepared to speak. They are sometimes connected with temperament or idiosyncracy ; but oftener with the Malaria itself, with the general condition of the atmosphere, or with previous, epidemic, constitutional tendencies, the offspring of undiscovered causes. Thus we have in one year known the first termination to prevail, in another the second, and in another the third. The fourth we have never known general ; but it has occurred, sporadically, with each of the others, and would, therefore, seem to depend on the constitution of the patient.

In the conclusion of this chapter our author has many judicious and interesting remarks on the diminution of the powers of intellect, apoplexy, palsy and neuralgia, as casualties occurring in the progress of the fever, or as modes of termination, the whole of which we recommend to the perusal of the reader.

Chapter II. is on the chronic, or relapsing and obscure, or anomalous remittent.

Dr. M. believes that many cases of what are termed by the nosologists, typhus mitior and synochus, and by the people 'nervous fever,' 'low fever,' 'slow fever,' 'fever on the spirits,' and so forth, are in reality the products of Malaria, and mere modifications of remittent fever. Still, he thinks, there *is* a contagious fever, distinct from that caused by Malaria ; and intimates that all fevers are perhaps referable to the two great causes, Malaria and Contagion. The reader will recollect that the late illustrious and lamented Dr. Armstrong, after assuming, throughout his work on typhus, the contagious origin of that disease, became convinced that it was, in fact, the offspring of Malaria, and published a recantation. Dr. M. however, with all his propensities to extend the dominion of this element of our ætiology, seems still to believe in the existence of contagion. Were we to depend on our own observations, only, we should say, that a contagious fever (not eruptive) does not exist—never yet having seen an example of it ; but on the other hand, having known autumnal fever to appear with most of the symptoms of mild typhus, when, of course, it was mainly produced by Malaria. We decline, however, the expression of a final opinion, on this difficult subject. Dr. M. suggests, as a diagnostic rule, that when a fever, with typhus symptoms, has a tendency to relapse, is curable by the means employed for the cure of remittent fever, and is attended with glandular, visceral affections, it should be considered, as originating from Malaria rather than Contagion.

But we must give our readers, in Dr. M's own words, a more circumstantial account of this obscure and relapsing remittent.

It will now be necessary to describe, as far as can be done, the symptoms of this fever, and most particularly when those are most slight ; since these are the very cases where erroneous observation is most common, and is followed by equally erroneous practice.

This disorder may be found, and not unfrequently, with scarcely any marked symptom except mere muscular weakness ; a debility on any attempt at exertion, which seems unaccountable, inasmuch as it occurs in persons, even in youth, and apparently strong, and is not very obviously accompanied by any proper febrile symptoms. At times, not even the appetite seems affected ; and here, almost necessarily, the result is, to suspect the state of the patient's mind, or his moral dispositions, rather than his health ; to suppose, for example, as I have often seen, that a soldier is "shamming," that an opulent female is indolent or affected, or a studious or professional man hypochondriacal.

Yet, let an acute physician watch this disease, and he will be convinced that it is a disease, and moreover a fever. It commences and terminates like the remittent when best marked ; and when it appears to be prolonged for months or years, as is sometimes the case, it will be easy to see that it has had intervals of cure, generally of self-cure, and relapse ; and that, to each relapse, there is a period of weeks, not very uncommonly of six, while the intervals vary from one or two to any given number. Further, either the patient or the physician, or both, must be very inattentive if they do not discover that the paroxysm of extreme debility is fixed ; that it is, in fact, a paroxysm, let its length be what it may, and that there is a diurnal period when it diminishes, or where the patient, who, possibly, could not stand, on getting up in the morning, is enabled to exert, and even to enjoy himself at night.

Hence, as to some cases, at least, the truth of, as well as the reason for, a very common remark, that midnight is the nervous patient's holiday ; though there are unquestionably many cases of nervous affection, and even of periodical returns and intermissions in this complicated class of disorders, which do not appertain to a remittent type of fever, or perhaps to any fever. The particular case here quoted, is one, of course, where the paroxysm attacks in the morning and the remission is at night ; but while the periods are necessarily various, so are the results, as to the complaints, appearances, or sufferings of the patient. I shall presently trace some others of the more marked of these modifications.

I have assumed here that pure debility may be the sole symptom of a remittent ; but it would have been more correct to say the sole obvious one, since it is rare but that the patient at least, if an attentive observer and a good reasoner, and if at the same time free from the morbid influences of the imagination, will not discover other indications of a febrile remittent disease ; however the physician may overlook them, from neglect, or perhaps from ignorance, or system ; or, as may also happen, from want of sufficient opportunities for personal observation. He moreover who would discover what he cannot see, by cross-examination, must know well what questions to put and how to present them ; or he may remain ignorant, from assuming a wrong course, or else gain the very answers which he has suggested. A leading question is too often as deceptive in physic as in legal procedure.

The obscure symptoms which I am now to point out are those, as might be anticipated, which, whenever they become marked, are also easily discovered ; and which, as they gradually multiply, and become also more conspicuous, indicate a more severe disease, gradually passing into a form so distinct, that the character of true remittent fever can no longer be denied to it, even by the most prejudiced. And these gradations of severity, if I omitted to adduce them just now as an additional argument in proof of the true nature of this disease, are, in reality, among the strongest evidences of its argued origin and cause ; since it would not be difficult for any attentive physician in tolerably extensive practice, to collect a series of his own cases, rising in exact gradation from the simplest debility to the most perfect and defined remittent fever.

I noticed that the appetite was sometimes not affected ; but, even in the slightest cases, a careful observation will show that it is partial and irregular, or, in common language, capricious. The fact in this case is, that it vanishes during the paroxysms, returning in the interval ; a term which I choose in preference to intermission ; while the common inaccuracy of observation as to this fact, or a diversity of judgment as to the condition of the appetite, arises from a coincidence, or the want of it, between the conditions of the fever and the usually established hours of eating. He whose paroxysm includes the hour of breakfast, may be unable to eat in the morning, while he can dine ; whereas a paroxysm extending till night, may make him suppose that his appetite has entirely vanished ; when, did he attempt to dine, or sup, at midnight, or at some period of the night, should that be the interval of health or abatement, he would cease to make this complaint. And thus it is also, that we meet with cases where the appetite seems unaltered amid considerable disorder ; because, in these, the paroxysm returns at night, and the days form the intervals. Hence a rule in the cure to be noticed hereafter, which relates to the hours of eating ; and, as will also be then shown, on similar grounds, which equally refers to those of sleep.

It is barely possible that in these slight cases, the condition of the tongue may be healthy, or nearly so, and that the *primæ viæ* may also be in a state of regular action. More commonly, both are affected in modes too familiar to require further explanation : but these, as belonging to so many other disorders, are seldom of any value as diagnostic symptoms ; not seldom also misleading a bad observer, and being considered the cause of disease, of which they are merely the consequences. In the whole catalogue of ordinary practical errors, I know few indeed more common than that which views a sluggish state of the bowels as a primary disease, sometimes also a consequence of theoretic disorders of the liver, instead of considering it what it really often is, the produce of a febrile state, belonging, either to this fever or to some other initiative and similar cause. Nor is it difficult to account for this error, vulgar as it is common ; since it is the consequence, partly, of seeing, in a disorder, nothing but obvious symptoms, and partly of that empirical practice for which England is so celebrated, and which, while it tends to blind the judgment, can, from it facility, be conducted by any one : while I need not tell the medical reader to whom we are indebted for its present influence and abuses ; an influence and abuses which, whether they are now increasing or diminishing, are convenient to indolence, by superseding the necessity of thought or investigation, and by reducing the whole practice of physic to an empiricism, to which I know not that its entire history can produce a parallel example.

I feel no hesitation in saying that fevers of this character are a very general cause of the chronic and common derangements in question ; and have no doubt that there are many physicians who are aware of this, and that many more will come to the same conclusion when they shall re-examine the disorders of this character under the present views. And consequently, that while these are symptoms of fever, not primary affections and causes of a febrile state, as is the common error under the system which I have been censuring, they may appear to constitute the sole disease, if the other symptoms are slight ; just as mere debility does in other cases : ready therefore to mislead, even an attentive physician, and much more certainly the cause of false judgments in those of a reverse character. In this fever, however, as is to be expected, such derangements become, in certain cases, more marked ; or, as the disease at large approaches to the more perfectly defined or more severe remittent, there occur all those circumstances which, as belonging to acknowledged fever, I need not dwell on.

What perhaps may appear most remarkable, is the state of the pulse ; and it is the symptom, above all others, which misleads unobservant practitioners. There are persons who cannot conceive a fever without an accelerated pulse : whereas, even in severe cases of remittent, the pulse often gives no indications of any disorder, or the very reverse of what such practitioners would have anticipated. At the very most, the periods during which the pulse is affected are sometimes so very transitory, that it is a chance if the physician should be present at the time ; while it is an observation seldom required from patients themselves, though, in such cases, perhaps improperly neglected. Thus it will happen, that in point of velocity, or of character in every way, the pulse may be natural during the far greater portion of the day, while for a period of an hour, or even far less, it may undergo that very peculiar change, consisting in asperity, or hardness, or diminution of size, or feebleness added to acceleration, generally, but not invariably, and sometimes indeed quite the reverse, which marks the commencement of the paroxysm, or the whole properly febrile state ; and which is all that we ever find of what may be called the cold stage, or of the hot one ; since these are, commonly, scarcely distinguishable in this fever, and since the former in particular can scarcely be said to exist at all in many cases. And if this state takes place in the night, as does, and not unfrequently, happen, in spite of the well-known remark that the majority of quotidian attacks of fever occur in the day, it may be as unobserved, or even denied, by the patient, as it is unknown to the physician. It is not therefore wonderful, should it happen, that, finding no proper or obvious febrile symptoms, hearing of nervous fever and nervous disease, and witnessing perhaps only nervous symptoms, dyspeptic ones, derangements of the bowels, debility, one, or more, or all, the practitioner who is influenced by a wrong system, or is without any views at all and is merely guided by terms, should conceive himself in possession of a "nervous" patient : acting accordingly, or doing nothing right, with perhaps a good deal that is wrong.

Here also I must remind the reader, that even in well-marked remittent fever, there is often a period of the day in which the pulse becomes slow, frequently falling below sixty ; while it is not unfrequently also full, as if under coma, and while this state is further attended by actual sleepiness approaching to coma, and not seldom, if this is not present, by lowness of spirits or melancholy. The same occurs in the slighter disease under notice, and in different degrees; while being also what is called a nervous symptom, it tends still further to mislead the negligent or ignorant practitioner; him who is guided by a correlative comparison of names and receipts, terms of diseases and the antagonist terms of medicines.

This may be thought a long extract, but we could not set forth our author's views in a shorter one. Even this, presents them but partially, for in several of the following pages he continues the subject, and treats of the morbid states of the feelings, passions, and intellectual functions, attendant on this obscure form of malarious disease. These are irascibility, peevishness, anger, fear, despair, transitory delirium, hypochondriasis, and even mental aleniation. In this brief catalogue the reader will recognize those affections of our spiritual part, which constitute the moral phenomena of dyspepsia; and, indeed, much of what is contained in the chapter, now under review, might with equal propriety be introduced into a monographic treatise on that malady. Thus, our author goes on to speak at large of lethargy, morbid vigilance, and disturbed and oppressive sleep, all of which are well known to accompany what is called dyspepsia, and to constitute some of its most distressing symptoms. The disinclination to early rising, from which no dyspeptic ever was or ever will be exempt, is looked upon by Dr. M. with a different eye, from that of most writers. He bears to the opinion that the patient should be indulged. He does not regard early rising as remedial, or even preventive. *He* is the early riser whom a sound constitution has destined for long life: it is not *because* he has risen early that he sees the borders of fourscore. To this opinion we subscribe an unqualified assent. Very few dyspeptics are inclined to sleep in the early part of the night, and we have not known one who did not feel wretched on awaking, especially if aroused early in the morning. The habit of late sleeping, which governs dyspeptics, is the consequence, not the cause of their disease. But to return. Dr. M. insists, that in numerous cases of what is called dyspepsia, an observing physician may find abundant evidence of the presence and actual workings of an obscure, chronic and relapsing remittent. If this be the fact, are we not furnished, with a key to the study of this protean disorder, in these western states ? We have long thought the generally acknowledged causes of dyspepsia in the West, insufficient to account for its prevalence ; and have believed and taught, that marsh miasma is one of its sources. We have not, however, pursued the subject in the manner, nor to the extent, of Dr. M. Nevertheless, we have observed, that dyspepsia is more prevalent in our alluvial villages, than elsewhere ; is frequently attended with febrile movements ; and, like a chronic malarious fever, will leave the patient, and recur upon him, without provocation, in the midst of his enjoyments. A malady of such habitudes, seems to have strong claims to admission among the diseases from Malaria ; and we, respectfully, commend the whole subject to the profession in the West and South, as one deserving of great attention.

Passing from dyspepsia, our indefatigable author goes on to speak of hysteria, as one of the modes in which remittent fever occasionally disguises itself ; after which he observes :

"Thus I have enumerated the leading and conspicuous symptoms of this modification, or of these modifications or varieties, of remittent fever, as far as they may be considered general ; reserving those which seem more properly of a local nature to another division of this subject."

"If I have now sufficiently described this modification of remittent fever, as I consider it to be, through its obscurer modes and symptoms, and if I have also given such distinctive characters as shall enable any one, with a moderate degree of attention, to recognize it, I have hitherto spoken of it as a definite disease, occupying the usual periods of others and more severe remittents. This was necessary for the sake of distinctness, and that it might the more easily be compared with the ordinary and acknowledged remitting fever : but it is a description that will seldom apply in practice, as far as the duration of the disease is concerned ; and, on this point, I must now proceed to offer some remarks.

Let me also here premise one observation, which, though already made, requires to be stated more distinctly and forcibly. This disease, in actual practice, is commonly of long duration, as I shall soon show ; while it frequently follows a severe attack of decided remittent, or an equally distinct one of quotidian or double tertian intermittent. It happens also that, according to season, or from the nature of the exciting cause of a fresh relapse, or from other circumstances, possibly not very apparent at present, it puts on rather the appearance of an intermitting than a remitting disease : the intervals, or intermissions, being perfect, perhaps long ; while, further, there may be a distinct cold stage, however short, at the renewal of each paroxysm. Thus might it equally have been classed under the head of chronic intermittent with the quotidian type ; though had I treated of it there, they who may now know it, or hereafter remark it, as a remitting disease without cold stages, might equally complain that I had misplaced it. There was but a choice ; and I have placed it in the division to which I have found it most frequently conform : while it is plain, that the dispute, should it arise, is not worth entertaining, inasmuch as the whole is but one disease under different modifications. It is, in short, the chronic febrile state of fever or fevers, which attend the unfortunate people who reside in the pestilential countries described in the essay on Malaria : putting on an endless variety of appearances, from which I have attempted to condense, as far as lay in my power, a general description adapted to the majority of cases, or at least capable of serving as a point of reference for disorders which differ in almost every individual. It is, in reality, the chronic marsh fever ; approaching, on one hand, to the acute and regular disease, and, on the other, to that undefined condition of the same nature which is so often called, simply, ill-health : while, as to type, it maintains the same gradation of character ; being continuous, in a limited sense, or remittent, or intermittent, just as are the severe marsh fevers to which it is affiliated. I am utterly indifferent where it is ranked, provided it be understood, for the sake of those who are its victims."

The remainder of this chapter is taken up with an account of some of the consequences of the obscure remittent fever ; with new illustrations of its character ; and a recapitulation of its leading symptoms. Our limits will admit of our copying but a few paragraphs. The following is a part of his recapitulation.

"It is a remittent fever, bearing all the characters of that disease as it is universally known ; but in a modified degree, and often so slight, as to require some attention in tracing its form and even its existence.

It is apt to become habitual, or to recur at frequent but variable intervals, during an indefinite course of years, so as even in some instances to occupy almost a long life. In such a course, it also varies its characters and symptoms, and in some cases, becomes a marked chronic intermittent ; while in others, the imperfection or brevity of the intervals may cause it to appear as a continued febrile state.

Its accessions, when they are to be defined, are as various in duration as those of the ordinary severe remittent.

It is the sequel, in some cases, of quotidian intermittent or of double tertian : and if a mild fever of this nature follows a common tertian, the length of the interval will oblige us to rank it under tertian, as a chronic disease. It is also the sequel of common and acknowledged remittent ; and thus it may also be a sequel of what is called typhus fever, because the remittent is often thus misnamed. It is equally a sequel of what is called low fever or nervous fever, which, equally mistaken for a mild typhus, is a remittent. And these also are the proofs of the real nature and origin of this disease ; since it is their continuation, or forms varieties under them. And, while its causes must be sought in Malaria, (though others are not absolutely excluded, in our present state of knowledge) even when it occurs as a primary disease, this also establishes, even further if that were necessary, its true nature.

Chapter III. is appropriated to the proximate cause of remittent fever. Of this subject our author candidly confesses his ignorance.

"Physic knows not how the poison of fever acts, nor on what it acts ; what are the preliminary effects which produce the symptoms which are obvious to our senses. It cannot even conjecture why these actions should cease, why they should be renewed, and with a regularity, often not to be exceeded by that of a machine, or why they should cease to be renewed.

Doubtless, the inquiry is a most interesting one ; but how can we inquire when we have not the slightest element ascertained, on which an inquiry can be founded ?"

Dr. M. does not go into a critical review of the various theories or rather hypotheses of fever, but selecting that of Broussais, holds the following language :

"The fever of marshes, say others, under whatever form, is a gastro-enterite ; or, essentially and radically, it is but an inflammation of the inner membrane of the stomach and bowels, one or all ; and every other effect and symptom is sympathetic or consequential : or "adynamique," arising from "sur excitation." On this is the practice founded, by those who thus believe in the last new theory ; while, it is said, the success of the practice, consisting in blood-letting and so forth, proves the truth of that theory. Let us inquire as to both : to say nothing of the evil results which flow from acting upon this fashionable hypothesis.

But first of all, how can such an inflammation, and of such a membrane as this, generally, is, produce so deadly, so universal a disease ; a disease so abounding in symptoms, present and subsequent, so endlessly varied, and so fatal ? The stomach, and the bowels also, are often much more and more extensively inflamed, and without such fever, or such symptoms, or such effects. In these fevers there is often no pain, not even on pressure ; and where then is the inflammation ? Thousands of persons die with all the symptoms of this fever, even within a few days, within a day, within an hour ; the gastro-enterite, here, ought to be excessive, yet there is none, for there is no pain. There is none, for a much better reason ; that it is not found on dissection. We may refer to a thousand authors, for thousands of dissections, where, if the bowels and stomach have sometimes been found inflamed, there has not been the slightest trace of present or previous inflammation in numbers without counting. And if this were not enough, we find patients who die of fevers with very slender symptoms, where there has been no pain ; and yet dissection proves the bowels to have even been seriously ulcerated. If inflammation had killed the patient in any case, how is it to disappear and leave no trace ? and what species of essential cause or symptom is that which is not always present ?

Whatever dissections have taught, they have not taught us the cause of marsh fever ; and let him who doubts, read all the monstrous volumes on this subject. They have taught us that certain effects take place occasionally : that is their use : and the gastro-enterites, the inflammations, be they what they may, are, in reality, symptoms or effects : how far they must regulate the practice, is a question not for this place : but let them regulate it in as far as they are such, not as being the essence and the proximate cause, and then they may effect good and not harm."

Not wishing on this occasion, to enter into a full examination of the doctrines of the French school, we shall proceed with Dr. M. to the cure of the fever which we have been considering. This is the subject of Chapter IV, and runs through more than 30 pages. Observing in it, nothing new or particularly worthy of being detailed to our readers, we shall not trouble them with many extracts.

Dr. M. in fact renounces all intention of treating of the cure of the remittents of hot climates. He commences by 'separating, in some measure, the treatment of the acute from the chronic remittent, or the simple from the relapsing or habitual disease, since there are some important differences in the practice.'

The first remedy which he considers is emetics.

"That they do occasionally terminate, or at least shorten the fever, must be admitted ; while they seem to be chiefly useful whenever the disorder is attended by an increased action of the liver, or in that variety which, with us, goes by the name of bilious fever."

Relying on our own observations and experience, we are constrained to say, that in the treatment of the remittent fever of this country, emetics are too much neglected. We have repeatedly seen them produce the happiest effects. If the force and fulness of the pulse be great, venesection should be premised ; and to obtain from it the greatest good, a full narcotico-diaphoretic portion should always precede their operation.

Both at the commencement and through the course of the fever, Dr. M. seems to sanction the use of cathartics, though his remarks are variously qualified. Throughout the whole disease, in his opinion, a state of the bowels approaching as nearly to the natural one, as may be, should be preserved. But this would imply the use of laxatives to preserve, not of purgatives to increase the peristaltic motion. Such a state of the bowels in remittent fever is moreover quite imaginary. When all the secretions are disorderd, how can natural evacuations, as to manner and matter, be expected ? They can only be obtained by curing the disease ; and when they occur the patient is convalescent. The question is, whether cathartics can contribute to this cure ? In the opinion of Broussais they cannot, but the reverse. If, however, they do harm, *ex necessitate*, how does it happen, that fever patients every day recover under their use, all over the United States ? Dr. M. prefers 'rhubarb, senna, &c. which act chiefly or solely on the natural evacuations' of the digestion organs, to the saline purgatives, which evacuate the entire system, and produce debility. It is true, that the latter, occasion more copious secretion, especially when united with antimonials, but on that very account are better adapted than the resinous cathartics, to the *acute* forms of fever, while for the same reason, they are prejudicial in the chronic and relapsing varieties; all of which are reproduced or aggravated, by debilitating measures. Of calomel Dr. M. is not an actual admirer, and believes that it is often abused; but when biliary derangements are manifest, either in acute or chronic remittents, he advises its use until the secretion of the liver becomes healthy, after which he thinks it does harm.

Sudorifics in the opinion of our author do but little good. Sweating and an abatement of the disease are often, perhaps generally, connected, but the latter is the cause not the effect. This on the whole is a sound opinion; and still we do not doubt, that promoting and maintaining a sweat, when it has begun to appear, may contribute to a more perfect solution of the disease. As a general rule opium should be combined with our sudorifies. When they are proper, it is admissible, and will contribute greatly to their effects. On this medicine our author has the following remarks.

"The use of opium requires also a few remarks, as it has been alternately lauded and condemned, chiefly from not attending to some very necessary distinctions. Generally, that it must be improper when it is necessary to administer purgatives, would be a natural conclusion; yet even here it is often useful, by facilitating the action of these from its antispasmodic effects; as it further is, by removing that irritation, which is apt to follow their salutary action.

As to its value in procuring sleep, though there may sometimes be even a state of drowsiness approaching to coma, and protracted also long through the disease, it is more common to meet with sleeplessness, or disturbed, or irregular rest; while I have even seen this consequence carried to such a degree, that the patient did not procure above an hour's sleep out of the twenty-four, during the whole period of six weeks which the disease lasted.

In all these cases, as long as the want of sleep is the produce of the febrile action, or attends the paroxysm, the use of opium serves but to increase the evil, as far as I have observed, or is productive of no advantage, at the best. But it does also happen, that during the remission, there remains an excitement, more visible in the faculties of the mind than in the body, preventing sleep; a condition under which opium is often advantageous, by procuring that rest which would not have come on till the new paroxysm was again ready to break it up. Thus also, as might be anticipated, it is more applicable towards the end than the commencement of the fever. With respect to its power as a stimulant remedy, whatever that be, its utility and application will be judged and guided by the same rules which regulate the use of wine."

On this subject we are disposed to express the opinion, founded on personal observation, that in remittent fever, opium is more safe and curative, than many physicians suppose. We have certainly known it administered freely throughout the paroxysm, with salutary effect. The tincture may be united with antimonial wine, or with the spirits of nitrous ether. The opinion that it might determine blood upon the brain, seems to us not well founded, provided depletion has preceded its use; while its power of calming the violent movements of the system, subduing its morbid sensibilities, and easing pain, entitles it to rank among our curative, not less than palliative remedies.

On food our author has, among others, the following judicious observations:

"In the most mild and chronic remittent, as was formerly remarked, the same bad effects, namely, increase of severity, or the protraction of the paroxysms, is a frequent consequence of the injudicious use of food There is but one period in which it can ever be given with safety, and that is, the interval, or remission; and, as I formerly noticed, the choice should fall on the commencement of that, while in the severer cases, where the distinction between the paroxysms is most imperfect, it is for the physician to watch the time when the least evil is likely to accrue, keeping in his mind these general principles.

And further, as, in many cases, this is a disease of debility, as it is indeed in all, after the commencement, it is plain that food, as far as the stomach can manage it, is even an important remedy."

The cold affusion, Dr. M. dismisses in a single paragraph. He thinks it adapted to the remittents of hot, rather than cold climates. We have found it beneficial in the fevers which are denominated typhus or continued; but are doubtful of its efficacy in remitting and intermitting fevers. Applied to the head, however, when affected with heat and pain, it often does good.

"If the use of bark, which may here stand as the representative of all the tonics, as it is that especially adopted, has been condemned by numerous and enlightened physicians in the proper remittent fever, there are not wanting as many who place confidence in it; guided sometimes by experience, and at others by habit, or by theories: just as is true of those who oppose its use. It is a complicated question; since, like every other one in this disorder, its utility or disadvantages will partly depend on the individual characters of cases, as of epidemics; not unfrequently on peculiarities in the patient himself, and on a variety of collateral circumstances as tedious to detail as they must be superfluous to medical readers. Without doubt, there are instances where, even though no inflammatory condition is present, it does not cure the disease, but aggravates, not merely the paroxysms but the entire disorder; whether, however, by any greater action than that of deranging and oppressing the stomach, appears uncertain. Even thus it may be pernicious: but I see no evidence of a very common opinion in physic, that, thus given, it is the cause of the glandular visceral diseases so common in these fevers, or so commonly following them. These occur equally where bark has not been given; they are often in existence even before the fever, and appear in many cases to be quite independent of it; while, ignorant as we are of their immediate causes or nature, we have no right to assume that they could be the result of a remedy with whose actions also we are equally unacquainted. This is one of the inconsequential and gratuitous conclusions in which physic abounds but too much; but it is one that is examined by evidence elsewhere, and on which I need not therefore now dwell.

The leading objection to the adoption of bark in this fever, is, however, derived from the presence of what is called inflammatory action, consisting in a high state of pulse and so forth, and very particularly from the presence of topical inflammation: and it is in these latter cases that it is so violently condemned by French physicians. Yet even here, opinions are divided; and it has often actually been found to be the remedy in such cases. It is as painful to a writer to leave his readers in suspense on points so essential, as it would be presumptuous to decide; yet it may be suggested that if, as will hereafter appear, the inflammatory affections of remittent are of a peculiar character, and not proper phlegmasiæ, and if certain visible and demonstrable ones are actually cured by this remedy, and aggravated by evacuants, the question will not improbably be decided in favour of those who recommend it in all cases; and it may not be difficult then to discover that prejudice or incorrect observation will explain that testimony against it which has been thought to be derived from experience.

The utility of bark or of tonics in general is, however, scarcely disputed in those cases where the symptoms are those of debility; and still more where they are what are called malignant; nor, in the advanced stages of a disease of this nature, even should the fever be almost continuous, or with imperfect remissions. Still less is it questioned when the length of the intervals and the perfections of the remissions have brought this disease into the congenerous class of intermittent fevers. As soon as that character is at all formed, this remedy may be used, and even by those who object to its general use, with the caution that it is not urged beyond the remission or intermission, if indeed that be necessary; and thus it often terminates at once a disorder, that might pass into a troublesome or chronic intermittent. For all else that belongs to its use, I may refer to the treatment of intermittents, hereafter discussed."

On the use of wine we extract the following observations :

" Theoretically, wine is held to be injurious, because it excites the action of the heart, and increases the tendency to inflammation, and because this disease is held to be of an inflammatory character. There are here two points involved ; and, to prove that wine must be injurious, it is necessary that both should be established. The question of experience forms a separate subject of consideration.

It may appear a very extraordinary doubt to suggest, against the almost uniform and continued opinion of physicians ; but is it proved that wine increases inflammation, when existing, or produces the tendency to it in healthy subjects or in diseased ones ? Will those who believe it ask themselves why they do so, and whether it is not one of the hereditary opinions of physic, established, no one knows why, and followed because it has been followed ? But, granting that there are cases of inflammation, or a species of inflammation which wine would increase, physicians know full well that they are utterly ignorant of the real distinctions among inflammation which, to the sense or the eye, may appear the same ; and that while there are some kinds or varieties which are to be cured by stimulants both local and general, as I shall hereafter show very fully, so are there inflammations, and apparently inflammatory states of the entire system with increase of circulation, where wine is a remedy instead of being injurious. Nor does it appear that the habitual use of wine produces a tendency to inflammation in healthy subjects ; since it is notorious that among water-drinkers, the diseases of active inflammation are most frequent, and require the most energetic treatment."

We come now to blood letting, a remedy, which most American physicians would place at the head of the means of cure in this disease ; for if not the most important, it is certainly one of the first in point of time. We shall make an extract of some length.

" That opinion is, that in the far greater number of cases occurring in our own country, the practice of blood-letting is most commonly nugatory or superfluous, or else injurious. But I must enter into some details on this subject.

In the first attack of the fever, and often through many successive diurnal periods, the pulse is hurried, contracted, hard, or full ; or the general appearance, to a common careless observer, is that of a state of circulation similar to what takes place in inflammatory affections. This state however is, when accurately viewed, the commencement or the first portion of the paroxysm, and its analogy is found in the similar state of circulation occurring in the first stage or stages of the intermittent paroxysm. And if it proves durable through any one diurnal period, or durable as it relates to the whole disease, it merely marks that condition of the paroxysm and of its returns, which equally occurs in certain cases of the pure intermittent. In other instances, it will be found that the duration of this state of the circulation is brief; or it subsides without the interference of the practitioner, as the paroxysm itself proceeds towards remission.

This fact and this analogy ought to explain what the real nature of this particular condition in the remittent is; and if it is so generally mistaken for an inflammatory state, perhaps the term inflammatory fever, and the apparently groundless erection of a disease of this character by nosologists, has aided in establishing an opinion which must however be chiefly traced to that servility of routine by which the unthinking mass follows the road which a predominant theory or a prevailing fashion points out to them as the easy one: since, of this quality, must the mass be every where.

Further, it may happen that there is also presents some topical symptom, such for example as headach, the most common of all. How often headach it the result of conditions the most opposed to local inflammation or inflammatory action, I surely need not say; but if, in the case under review, it exists with this particular state of the circulation, it is easy to see how, under the previous theory or prejudice, it will be conceived to depend on a fixed topical affection of the brain. Thus also is a casual catarrh, not an unusual accident in this disease, attributed, under the same prejudice, to inflammatory action in the lungs; and thus even does it occur frequently, that such a combination is actually mistaken for pleurisy or peripneumony. The same reasoning applies to other topical affections, and I need not therefore dwell on it; besides which, as I have already suggested in speaking of bark, and shall hereafter explain more distinctly, the predominant, I do not say the whole of the topical inflammatory affections in this disease, are of a peculiar character, and not true phlegmasiæ. And, if I wished for a very high authority of a somewhat distant date on this point, as I have, in speaking of intermittent, alluded to many modern ones, I would refer to Lobb, who decidedly rejects blood-letting in fevers of this nature, however violent and inflammatory the symptoms; and even in the cases where acute pleurisy or rheumatism are present, or in the diseases so often considered as pure pleurisy and pure acute rheumatism. It is not, however, for me to deny that real inflammations requiring the lancet, may exist or have existed in recorded cases that have not come under my own recognisance.

It is easy therefore to see the causes which, united to a prejudice, or in hands governed by a routine, lead to that which I here consider an erroneous judgment; and I may now inquire what the effects of blood-letting really are; since, although in the majority of cases, it is, as commonly practised, injurious, as there are others in which it is without important effects of any kind, there are a few in which, under restrictions, it appears beneficial.

In a robust patient, at the first attack of the disease, and when the fever is the first of its kind to that patient, blood-letting is often useful, and at least not injurious. Its good effect is that of reducing that activity of the circulating system, or that vigour generally, which renders the first portion of the paroxysm correspondently energetic; thus moderating the whole result, not only of the first, but also of the subsequent paroxysms, and consequently of the total disease. And to judge respecting the propriety of its repetition, we must be guided by the same rules: a consideration of the constitution and character of the patient, and further, not merely of the appearance of the disease in that patient, but of the prevailing character which the endemic of that particular season may possess: as it is most certain that in different seasons, the remittent does vary most essentially in its character."

This subject is one of deep interest to us all. We are convinced that the fevers of some autumns do not call for the lancet; and are even made worse by its employment; while those of other years cannot be successfully treated, if it is neglected. We doubt, however, whether the autumnal fever of the United States, is ever, strictly speaking, cured, by blood letting, as it cures a pleurisy or peritonitis. Still, it contributes to preserve the life of the patient, and is, therefore, an important remedy. In many cases it is a *sine qua non* to his recovery, not by shortening the disease, but preventing its destroying life. Of course it is most requisite in the sanguine and plethoric, whose vital organs are most apt to suffer from a great volume of blood, put into violent motion, by the morbid contractions, a powerful and irritated circulatory system. In such cases, a copious blood letting, which may even be repeated once or twice, employed in the early periods of the disease, and during the increase or achme of the hot stage, may produce every other beneficial effect, except preventing the return of the paroxysm. In these cases we should bleed, for nearly the same reason, that we bleed in the eruptive fever of small pox—not to cure but to mitigate the disease. The latter is seldom accomplished. The malady, once established, will in general hold on its course, for, like many other morbid states, it has a period of its own, which may vary in duration exceedingly, it is true; but within which, certain morbid changes must be passed through, as in measles or the artificial disease, termed narcotism by the French, which comprehends the morbid effects of a large dose of opium. If the fever be violent, its course will be short; if mild, more protracted. Blood letting (except in the first paroxysm) can only moderate the former, and cannot cure even the latter ; in the treatment of which it is, in general, not required.

In epidemics, when the tendency is to terminate by a crisis, at or about the end of the first week, blood letting is in general most necessary and beneficial ; in those which are prone to assume an intermittent type, it is less needed ; in those which tend to a typhus character, it is often prejudicial. We are aware, that many physicians believe, that the typhus stage may be prevented by blood letting, and occurs from its omission ; but others have observed, that in certain epidemics, no resort to the lancet will ward off that perplexing and dangerous stage ; and that it will even be more certainly induced by copious depletion, especially in the advanced periods of the remittent. The fact seems to be, that it is the inherent tendency of such epidemics to become typhoid, which the lancet favours rather than prevents.

As to blood letting in the fevers termed congestive, which since the dissemination of Dr. Armstrong's work on typhus, have become so prevalent in the West and South, we are not prepared to speak decidedly. The central organs in these cases are inundated with blood, at the expense of the circumference, and if they cannot be relieved but by blood letting, it should be employed. We ought never to forget, however, that debility of some sort, the immediate effect, perhaps, of the remote cause, is, itself, the cause of these internal accumulations ; and that as blood letting is, in its nature, a debilitating measure, it *may* do harm instead of good. In full habits, however, it is no doubt admissible and even necessary ; though we cannot believe, that its repetition is in general either requisite or safe.

On the subject of local blood letting, Dr. M. says but little ; much less, indeed, than the fashionable—perhaps intrinsic—importance of this remedy calls for. On cupping and leeching over the epigastrium, he is silent, though we are compelled to believe, that it is one of the remedies for remittent fever, on which reliance should be placed. Whatever may be the difference of opinion in the profession, as to an original gastroenteritis, in this fever, it is undeniable, that in its progress the epigastric viscera, are liable to suffer deeply; and to their relief, few things are so well adapted, as local blood letting. We consider it a matter of regret, that throughout all the interior of the United States, every where indeed out of the large cities, our physicians, are unable to resort to leeching, and at the same time, most of them are unprovided with a cupping apparatus, and unpractised in its application. For the latter deficiency there is no excuse, whatever apology there may be for the former. In our own practice we have certainly derived from epigastric cupping, advantages which have appeared to us unattainable by any other means.

Not remotely connected with this subject is blistering. Of this remedy Dr. M. does not speak in very favourable terms. He informs us that he witnessed—

> "In a hospital practice of great extent, and through a long course of years, the invariable use of blisters in these cases by one physician, and the as invariable neglect of them by the other, in equal attendance; when the results, as proved by the registers, were absolutely equal for both, during the whole period."

This is certainly an important statistical fact, which we take the more interest in disseminating, because we know, that in the western country, there are many, otherwise judicious, physicians, who subject their fever patients to unmitigated blistering, a practice which has long appeared to us equally unnecessary and cruel.

The close of this chapter is on the cure of the chronic obscure and relapsing remittent. For this purpose the bark and arsenic might be supposed useful, but Dr. M. thinks them of little value; and, indeed, reposes no confidence in any medicine.

> "I know not that I should be very wrong if I were to commence by saying, in an equally sweeping manner as of the simpler modification of remittent last discussed, or of the simple fever without returns, that it is incurable by medicines, or beyond their power. I know of no great average of instances at least, in which the returns or relapses have been prevented by mere medicines, or where those seem to exert any influence for good, over the paroxysms of each relapse, or over the total state of a whole relapse. The inveterate career of this disease appears to be equal to its obstinacy of character in each recurrence.

If however the relapses be severe, the same rules apply as to the single disease, and those I need not repeat; but that relapse past, no remedy, no medicine at least, appears to prevent the habitual and expected return; or the returns are the same in point of period and force, where medicines have been used in the interval and where they have not. And it is in this disease that bark, arsenic, and the remainder of the tonic remedies, seem to be peculiarly without power; and not less so when the intermissions during the relapse, or the commencement of each paroxysm, are so well marked as to leave no hesitation in referring it to the quotidian and double tertian rather than to the proper remittent.

This at least is the result of my experience, and pretty generally of my reading also; but I shall be very well pleased to be contradicted by any one who may have had more success with medicines; as there is perhaps no disease in the whole catalogue of human miseries, attended with greater suffering, and not one with equal ininconvenience. To spend years of life in the sufferings of fever, sufferings which a spectator cannot appreciate or even comprehend, indefinite as they are, and unmarked by visible disease or by absolute pain, however well known to those who have felt what fever is, might be even misery enough, comprising as they do, bodily evils as unceasing as they are numerous and distressing. Add to this, nights resembling the days, sleeplessness, often with that delirious activity of mind which aggravates the present as it anticipates further evil, which longs not to think, but is compelled to be ever thinking: add to it the consciousness of ability for mental exertion, yet the perpetual torment of being forever checked in the attempt, the utter insensibility to every pleasurable feeling or impression, the dark present and the darker future, the "lasciate la speranza" which is so highly characteristic of this disease; add to it again the persevering coma, where the patient cannot rouse himself, yet cannot sleep, and a slight idea, slight indeed, may be formed of this tormenting disorder; less known, fortunately, in our happier climate, than to those pestiferous regions described in the first part of this essay, in which life is, most literally and truly, one long disease, and where death is indeed the only physician."

It is on change of situation only that reliance can be placed.

"Of what is in our power, if it is always just to the patient to labour through the whole round of tonics, with whatever little hope, there is nothing which seems really efficacious but change of habits; change of air, change of climate, change of every thing. And thus is the disease sometimes extirpated. But the value of this remedy is greatest when that change is from a soil productive of Malaria, or from any other situation generative of the causes of the disease; for by this is it often kept up, when it would otherwise disappear. Thus, as I formerly hinted, is it often supposed to be an habitual disease of the constitution, or even a disease dependent on organic affections, when, in truth, it is not a disease of relapses, but a succession of new diseases, produced by repeated applications of the cause."

In proof of the great importance of a change of place, Dr. M. cites the following important facts.

"In the first example, out of sixteen men from a frigate, under the African fever and in the hospital, eight were taken on board and to sea, the other half being left on shore; the consequence being, that all the former cases recovered perfectly and without relapse, while every one of the remainder subsided into an obstinate intermittent. This is a pointed case on a small scale. A recent and a very conspicuous proof, on a far greater one, of the utility of removing the convalescents from fevers to a healthy district, occurred at Rangoon in the late Birman war: the troops, in great numbers, which had continued to be sick and incapable of duty for many months at that place, after the fever, having recovered immediately and perfectly on their removal to Mergui."

After citing some other examples, our author goes on to remark:

"Here then we see more distinctly, the value of the remarks on pernicious soils and on the propagation of Malaria, which have been detailed in the essay on that subject. It is this study which must form the real basis of the cure, in this as in all the chronic or repeated intermittents. The cure consists in the avoidance of fresh causes, new excitements: and, to be enabled to apply it, these must be familiarly known, accurately studied, and carefully investigated. He who would be the real physician here, must be the acute observer and the philosophical naturalist; for where he cannot act he must learn to avoid, since the cure is negative, and in avoidance is the remedy."

The conclusion of this chapter is on the treatment of those organic, visceral affections, which are connected with the chronic and relapsing remittent. The only medicine of which he treats is mercury.

"On the presumption that the chronic fevers of this class were necessarily connected with organic diseases of the visceral glands, this remedy has naturally been recommended and followed. That it does act in the chronic inflammation of the liver as in the acute one, admits of no doubt: but there are many physicians of far greater experience than myself, who doubt with me of its power over the other chronic diseases, the real permanent diseases, or the obstructions as they are called, whether of the liver or the spleen. But this is a subject by no means so clear, difficult as it is to discover what the exact state or nature of the disease in these glands may be, as to prevent us from trying a remedy which is often doubtless a powerful one. That it is a practice often rashly and injuriously pursued in the present day, and especially in England, is however but too plain; while it is easy to trace the origin of this pernicious abuse to the physicians of our colonies, and especially to opinions formed and fostered in India."

Our author continues his strictures on the use and abuse of calomel in these disorders for a page or two, when he comes to say:

"On the other hand, now, so intricate is physic in every point, there can be no question that in this assertion there is some truth, or that there are cases in which mercury does most clearly produce a cure. That it has produced such cures while it also removed glandular affections, there is no doubt; while it is further possible that the cure of these disorders may have been the removal of the causes of the fever. But it is certain also, that it does cure this disposition to the renewal of fevers, even when there is the most perfect demonstration of a healthy state of all the viscera. In this case it seems to act by inducing a new disease, or an entire change of habits; just as acute diseases of various kinds remove it, or it is removed by any other important change in all the habits. Thus have I seen habitual fevers of this nature gradually disappearing under the use of mercury, while it was easy to trace the progressive connexion between the effects of the remedy and the diminution of the disease."

Without professing or feeling exactly the same dread of mercury, in these disorders, as our author, we do not hesitate to express the opinion, that in this country, it is often prescribed in organic affections of the viscera, both abdominal and thoracic, when it should be withheld. Not that we would entirely prohibit its use. In minute doses, both calomel and the pil. hydrargyri may be continued for several days with advantage; and an *occasional* cathartic portion, will often afford that relief, which no other remedy can give. What we object to, is a liberal or protracted use of these medicines, under the good old indefinite term alterative, until they excite a debilitating mercurial irritation, which cannot cure, and seldom fails to make the patient worse.

*Dysentery, Diarrhœa and Cholera*, are the subjects of Chapter V. The main object of our author, is to show that they have a common origin with Remittents; but in expressing his doubts, whether they ever come from intense heat acting on the body, as many writers suppose, he does not undertake to assign a reason, why, at different times, malaria can produce diseases so unlike as dysentery and ague.

The following paragraph presents facts, which are, certainly, not applicable to this country.

"If heat alone were the cause of cholera it should be produced in the earlier parts of the summer, supposing that such summer had been both early and warm; or if it required one month, or two, of the sun's action to generate this derangement of the liver, it should be indifferent at what part of the year that was exerted. The same argument indeed applies to the case of remittent; equally, and probably under the same error, supposed by many physicians to be the produce of mere heat. But, in practice, cholera, as well as remittent, is a disease of autumn; or it appears together with fever and dysentery, after a long, or a certain, duration of heat, and when, as is now admitted in as far as remittent and dysentery are concerned, some change has taken place in the vegetating surface of the soil, to which is owing the production of Malaria."

From observations continued for nearly thirty years, at Cincinnati, we are enabled to state, that cholera and diarrhœa do not appear, simultaneously, with remittent fever; but in general, precede it; invariably commencing before the solstice, while fever as constantly sets in after that period. In fact, they are the offspring—direct or indirect, which we do not pretend to say—of the first intense heat, and, therefore, often commence by the 20th of May; while, in other years, according to the development of great heat, they are postponed to the latter end of June. Moreover, we have known them nearly suspended by the occurrence of cool weather, and seen them recommence with its cessation. Nearly the same remarks are applicable to dysentery, though, on the whole, it is of later occurrence, and is, therefore, often associated with remittent fever. The natural order of succession seems to be, cholera and diarrhœa, dysentery, remittent fever, ague: the two first in early summer, the third at mid-summer, the fourth in its decline, and the fifth in autumn.

Having as he conceived, assimilated the intestinal affections just mentioned, to remittent and intermittent fevers, our author comes to speak of the last, which makes the subject of the next four chapters of his work.

He sets out with the following declaration concerning the cause of this form of fever :

"That intermittents, of whatever type, are the produce of Malaria, is a fact as universally established as any thing in medicine can well be ; while, as I have formerly observed, it is by no means proved that there is any other cause, at least of the original disease, or first attack, however such causes may have the power of re-exciting an intermittent where it has once existed."

The remainder of the chapter from which this paragraph is extracted, comprises the history of simple intermittents, and we shall notice it no further than to make the following excerpt :

"Thus, and for obvious reasons, it is also a general rule, that the vernal diseases, being intermittents or otherwise, are more inflammatory ; while the fevers of autumn are more generally malignant, or have the characters attending debility, together also with a greater predominance of bilious affections. And if we proceed along the season in the same manner, we shall find that while remittents continue in their proper form during the hot weather, they begin to intermit, even in hot climates, on the setting in of the cold. A natural conclusion which follows this, is, that be the power or effect of Malaria what it may, or however its production may be regulated by the state of temperature and the corresponding conditions of vegetation, there is, in the mere action itself of heat on the body, an accessary cause which is of considerable effect in determining the form and character of marsh fever. The well-known rarity of simple and original intermittents near the equator, must therefore be supposed to depend on these different circumstances combined. That, of the different types, quotidian and tertian are the general intermittents of spring, and quartans those of autumn, is a remark as old as physic itself."

Chapter VII. is on anomalous, obscure and simulating intermittents. In the beginning we find the following general observations.

"Let me therefore now remark, that in remittent fever, there may be present, inflammations of the brain, of the lungs or the pleura, of the throat, (anginæ of different characters,) of the bronchial membrane (catarrhs, of the stomach, the intestines, the liver, the spleen, the peritoneum, perhaps of other internal organs or viscera, and also of the muscles and ligaments (rheumatisms :) all of these varying in their intensities, in whole epidemics as in individuals, and thus, at one extreme, producing cases, which, under peculiar characters also in the essential fever, may simulate, or almost equal, cases of phrenitis, pleurisy, and so forth ; calling for material variations in the practice, and not unfrequently becoming the grounds of most inconvenient disputes among physicians not sufficiently aware of the nature of what they had not witnessed ; as also of recommendations as to the treatment, not less easily misapplied, and frequently highly pernicious in their results.

Now if these local affections occur in remittents, or, as must equally happen, in marsh fevers of a continuous character, sometimes even leading to error as to the fundamental nature and cause of the disease, or passing for pure inflammatory diseases whenever the physician is unaware of the nature of the epidemic, or when its true character has not yet betrayed itself, so they occur also in the severer intermittents, as might, without much effort of thought, be expected. In these cases perhaps, the varieties are not only greater, but error is even more easy. The inflammatory, or local affections, be they what they may, may be slender and truly supplementary ; but they may also prevail so far above the fever, as in remittent, that they may appear to be a distinct disease, or the superior one ; while if, further, the local affection should be permanent or continuous, when the fever has its intermissions, and perhaps long ones, as in quartan, and while, still further, that fever may not be very conspicuous compared to the local symptoms, it is easy to see that erroneous views and erroneous practice may follow.

Though I have thus referred only to the inflammatory local affections, as the most conspicuous in deducing this resemblance between the varieties of remittent and intermittent, the same analogy holds as to other variations or combinations, among which the nervous diseases, to use a popular term, form the great mass ; or, as there is a pleuritic remittent and a pleuritic intermittent, so apoplexy or palsy may be united in different modes to either mode of marsh fever. Such diseases, or such varieties of intermittent, will here be enumerated as far as they are known to myself, and also where, having never met with them, I can borrow from valid authorities ; but I must now state, generally, the reasons why I have made of this subject so extensive and detailed a chapter, and how far what I have described, differs from what will be found in authors on intermittent fevers."

After these and other general introductory remarks, Dr. M. comes to treat of apoplexy as an accompaniment of certain intermittents.

"I formerly noticed the apoplectic state which sometimes commenced the attack of intermittent as well as of remittent ; but it is necessary to recur to it here, on account of its great importance as connected with the practice in these disorders, and because of other analogous symptoms sometimes occurring. This state is not limited, either to the first attack or to the acuter forms of these fevers, although in Italy that appears to be the most common mode of its occurrence; as I have seen, in this country, a perfect apoplexy, to the eye, in all its characters, sufficient to deceive both the attending practitioner and the friends, lasting for eight hours, and occurring in a chronic tertian of many years standing, as a substitute for the cold fit."

Our author considers it important not to confound this state with original apoplexy, inasmuch, as the chief remedy of that disease, blood letting, is in his opinion extremely mischievous here. A modified condition of this symptom is lethargy or coma, which may either usher in an intermittent like apoplexy, or attend on its chronic forms. Our author then proceeds to speak of palsies. These are sometimes the inevitable consequence of the intermittent, but oftener in his opinion the effect of treating the apoplectic or comatose state by blood-letting. They are of course not to be cured by that remedy, which often renders them permanent, when they would otherwise be transitory. Dr. M's views on this point are sufficiently set forth in the following paragraph.

"The practical error in the case of such paralytic affections, is the same as that in the former two conditions; the imagining a "flow of blood to the head," and a palsy to be treated by bleeding and evacuants; and the result very often is, perhaps in severe attacks always, to confirm as a perpetual palsy, that which would have passed away of itself, had there been no interference, or which would have been removed by the common remedies for intermittent. If there are many other cases of palsy occurring in practice, which might otherwise have been temporary, whether arising from the effects of cold or not, and which have been rendered incurable by this pernicious and common treatment, I have little doubt that innumerable ones will be found, by those who have the means of making the inquiry, where this result has been the consequence of such evacuations applied in the palsy of the intermittent."

On the subject of intermittents and palsies, Dr. M. has an extended series of observations and reflections, to which he attaches great importance, as appears from the following sentence:

"So important do I consider this view of paralytic diseases, and so entirely have I found it neglected, even by modern and recent writers on palsy, as well as by writers on intermittent, that I almost wish I dared take room for such cases as might serve to make a deeper impression than what I ought perhaps to expect from this general statement, strongly as I wish to urge it by thus dwelling on it."

In many instances these palsies are strongly pronounced.

"But besides the more palpable and sudden paralytic affections attending intermittent, and under whatever modification or in whatever stage of the disease they occur, there are others of a more gradual nature which it is necessary to point out, not merely from their bearings on the practice in this disease, but from the light which they throw on the nature of intermittent, and very particularly on the other local affections belonging to it. If the variety in the effects or the appearances may be considerable, regulated as these must be by the nature of the nerves affected, and by the extent of the affection, it will be sufficient to describe one of these cases, and that account will perhaps also form the most convenient description.

This was an instance of a relapsing or chronic quotidian, in which there had, at no previous time, been observed any affections of this nature. During the relapse in question, which lasted the more general time of six weeks or two months, there came gradually on, a feeling of weakness in one leg, and more particularly in the foot, which increased so much during the disorder, that the patient could with difficulty make a step on that foot. And although there was no pain, or Neuralgia, it was easy to trace with the finger the course of the fibular nerve from the middle of the leg into the foot; as the slightest passage of the finger over it was attended by the well known tingling sensation produced by a pressed nerve; while a stronger pressure where it is nearest the surface, gave the equally well-known shock produced by striking the elbow, in another superficial nerve. It was obvious that the muscles chiefly affected in this case, were those to which this nerve principally belonged, and therefore that it was in a diseased state; that derangement appearing to consist in a diminution of its energy or power, added to an increased or morbid sensibility. The analogy of this to the proper Neuralgia is evident; and it must be considered, in fact, as a modified degree of this disorder, offering one of the proofs, out of so many, respecting the real nature of this disease, or its connexion with the intermittent and Malaria.

In this case, the relapse now described was succeeded by one much more severe, and successively by a third, more serious still. In these, the affection of the nerve of the leg, which had become little noticed during the interval, returned with increased severity; while, in the first, there came on a similar affection of the ulnar nerve of the right arm, and in the second, a similar one of the left; the others remaining. In this, most complete attack, the courses of the nerves in both arms could equally be traced by the finger in the same manner; while, further, the patient was unable to extend the arms without immediately feeling the tingling, as of a compressed nerve, along the whole course, from the arm-pit to the fingers; that sensation being increased by increasing the extension. And in this case also, it was evident that the muscles chiefly supplied from those nerves were the ones affected ; being feeble, or occasionally benumbed, as in the leg. Whatever be the exact affection of the nerve in this case, it is plain that it must be of a local, and, if I may use such a term, of an anatomical nature ; or that it was one which might possibly have been ascertained by dissection and inspection, had that been possible, and were we sufficiently acquainted with the intimate or ultimate structure of a nerve.

I need only further add respecting this case, that while the nerves of the arms recovered, yet very slowly, after the cessation of the fever, that of the leg remained diseased for many years after, and perhaps continues so still ; extremely sensible on casual pressure, even as far as the toe, so as to render great precaution necessary in walking, while the debility of that leg remained equally marked. If we see here the resemblance of this condition to that which succeeds to decided Neuralgia, so is its resemblance to the effect of sciatica on that particular nerve equally obvious ; while I propose hereafter to consider sciatica, in all cases, as a pure and perfect Neuralgia."

Having spoken of apoplexy and palsy as casualties connected with intermittents, our author, in following out his subject, is led to enumerate and illustrate, a great number of consequences of intermittent fever, especially when they become chronic. In the first place he remarks :

"It is a noted fact, that it is the effect of chronic or habitual intermittents to injure or destroy the intellectual faculties, as I had occasion to point out already when treating of remittents. This is notorious in the countries where these disorders prevail, and very remarkably, as I formerly said, in the Maremma of Tuscany, where even absolute idiotism from this cause is common ; the fact being marked, even to cursory travellers, by that apathy, listlessness, or indolence of mind, gradually approaching to fatuity, which I formerly described. If the cause be obscure, it cannot well be more obscure than every thing else which belongs to the action of Malaria ; while the fact of the universal influence of this poison on the nervous system, local as well as general, leaves no difficulty, at least in believing that it may produce such effects on the mind." D.

[*To be continued in the next Number.*]

